# Myocardial injury and cardiovascular complications in COVID-19: a cohort study in severe and critical patients

**DOI:** 10.5935/0103-507X.20220440-en

**Published:** 2022

**Authors:** Ana Palmira L Neves, Mauricio Nassau Machado, Joelma Vilafanha Gandolfi, Luana Fernandes Machado, Juliana Devós Syrio, Graziella Luckmeyer, Suzana Margareth Lobo

**Affiliations:** 1 Intensive Care Unit, Hospital de Base, Faculdade de Medicina de São José do Rio Preto - São José do Rio Preto (SP), Brazil.; 2 Department of Cardiology, Faculdade de Medicina de São José do Rio Preto - São José do Rio Preto (SP), Brazil.

**Keywords:** Myocardial injury, Myocarditis, Cardiovascular complications, COVID-19, Coronavirus infections, SARS-CoV-2, Critical care

## Abstract

**Objective:**

To characterize myocardial injury and cardiovascular complications and their predictors in severe and critical COVID-19 patients admitted to the intensive care unit.

**Methods:**

This was an observational cohort study of severe and critical COVID-19 patients admitted to the intensive care unit. Myocardial injury was defined as blood levels of cardiac troponin above the 99th percentile upper reference limit. Cardiovascular events considered were the composite of deep vein thrombosis, pulmonary embolism, stroke, myocardial infarction, acute limb ischemia, mesenteric ischemia, heart failure and arrhythmia. Univariate and multivariate logistic regression or Cox proportional hazard models were used to determine predictors of myocardial injury.

**Results:**

Of 567 patients with severe and critical COVID-19 admitted to the intensive care unit, 273 (48.1%) had myocardial injury. Of the 374 patients with critical COVID-19, 86.1% had myocardial injury, and also showed more organ dysfunction and higher 28-day mortality (56.6% *versus* 27.1%, p < 0.001). Advanced age, arterial hypertension and immune modulator use were predictors of myocardial injury. Cardiovascular complications occurred in 19.9% of patients with severe and critical COVID-19 admitted to the intensive care unit, with most events occurring in patients with myocardial injury (28.2% *versus* 12.2%, p < 0.001). The occurrence of an early cardiovascular event during intensive care unit stay was associated with higher 28-day mortality compared with late or no events (57.1% *versus* 34% *versus* 41.8%, p = 0.01).

**Conclusion:**

Myocardial injury and cardiovascular complications were commonly found in patients with severe and critical forms of COVID-19 admitted to the intensive care unit, and both were associated with increased mortality in these patients.

## INTRODUCTION

Severe acute respiratory syndrome coronavirus 2 (SARS-CoV-2), which can lead to coronavirus disease 2019 (COVID-19), spread rapidly worldwide and was declared a pandemic by the World Health Organization (WHO) on March 11, 2020.(1) Globally, as of February 11, 2022, there had been 404,910,528 confirmed cases of COVID-19, including 5.783.776 deaths, reported to the WHO. As of February 7, 2022, a total of 10,095,615,243 vaccine doses had been administered. SARS-CoV-2 is an RNA virus of the *Coronaviridae* family that can affect several organs, including the respiratory system, kidneys, gastrointestinal system and cardiovascular system.(2,3) COVID-19 has an incidence of approximately 15% of symptomatic cases and includes patients with pneumonia and hypoxemia needing hospitalization. In 5% of cases, there is a severe or critical form with respiratory failure requiring ventilatory support or shock and other complications, such as coagulopathy, thrombotic complications, bleeding, cytokine release syndrome, shock, and multiple organ dysfunction.(4,5) Genetic variants of SARS-CoV-2 have emerged and circulated in different parts of the world since the beginning of the COVID-19 pandemic. However, toward the end of 2020, several novel variants with superior transmission potential and infectivity were reported, which are associated with a severe form of the disease. Some of the variants possess superior transmission potential, altered pathogenesis and disease severity and are linked to the rapid increase in COVID-19 cases and associated with hospitalization and higher mortality. Currently, the Omicron variant is the variant of concern. Data show that it spreads more easily than other variants and is less severe in general. However, a surge in cases may lead to significant increases in hospitalization and death. Additional data are needed to fully understand the severity of illness and death associated with this variant.^(6)^

The cardiovascular system is broadly affected by SARS-CoV-2 infection, both directly and indirectly. SARS-CoV-2, like other coronaviruses, uses angiotensin-converting enzyme 2 (ACE2) for cell entry.^(4,5,7-11)^ Direct viral infection and indirect injury resulting from inflammation, endothelial activation, and microvascular thrombosis occur in COVID-19. A fifth to a third of hospitalized patients have evidence of myocardial injury, defined as high cardiac troponin (cTn) levels at admission.^(4,5,9-14)^ In severe cases, SARS-CoV-2 progresses from ACE2-dependent alveolar damage and hypoxia to systemic inflammatory response syndrome, acute respiratory distress syndrome (ARDS), and an exaggerated release of cytokines. The result can be a myocardial supply and demand imbalance, plaque rupture, and thrombosis due to procoagulant states.

It seems that the extent of cardiovascular injury is determined by the amount of viral inoculum, the magnitude of the host immune response, and comorbidities. The complications and lethality rates of COVID-19 vary in different countries, and the impact of myocardial injury and cardiovascular complications on outcomes is not well defined.^(15)^ We hypothesized that the presence of myocardial injury and cardiovascular complications could be related to the severity of the inflammatory response and other organ dysfunction and would impact outcomes. We aimed to characterize severe and critical COVID-19 cases with myocardial injury and cardiovascular complications as well as the predictors of their development.

## METHODS

This single-center, observational cohort study was performed at the *Hospital de Base* of São José do Rio Preto City, São Paulo, Brazil, a tertiary university hospital and designated center for treating patients with COVID-19.

A retrospective analysis was performed of data collected from March 25, 2020, to November 24, 2020, from all consecutive patients with suspected COVID-19 admitted to the intensive care unit (ICU). The study was approved by the local Institutional Review Board (CAAE: 31725720.2.0000.5415) and followed the Strengthening the Reporting of Observational Studies in Epidemiology (STROBE) guidelines.

The primary objectives were to investigate the proportion of myocardial injury and cardiovascular complications in severe and critical patients with COVID-19 and their characteristics and predictors. The secondary objectives were to study clinical and laboratory findings, particularly inflammatory markers, in severe and critical COVID-19 patients with myocardial injury and cardiovascular complications. Severe COVID-19 patients were those with clinical signs of pneumonia and one of the following criteria: respiratory rate > 30 breaths/minute, severe respiratory distress, and/or oxygen saturation (SpO_2_) < 90% in room air. The critical COVID-19 patients were those with ARDS or respiratory failure requiring ventilation, sepsis, or septic shock.^(16)^

**Figure 1:**
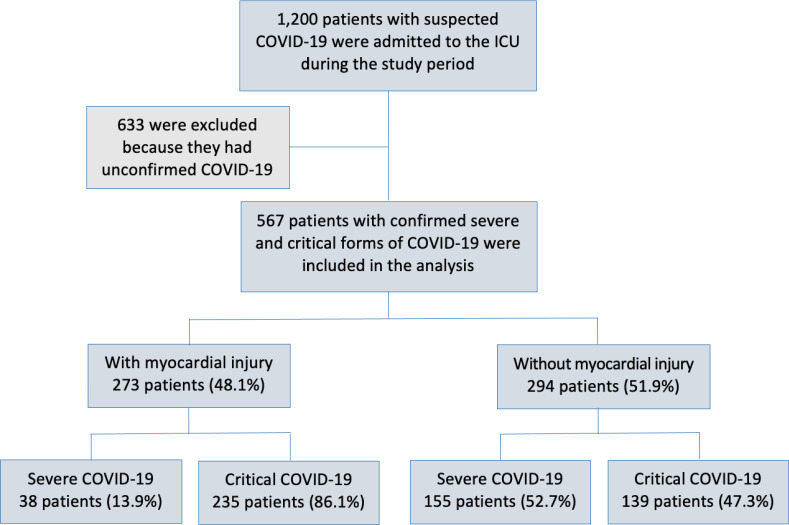
Study cohort.

The inclusion criteria were patients admitted to the ICU, over 18 years old, with confirmed SARS-CoV-2 infection by a positive result on polymerase chain reaction (PCR) testing of a nasopharyngeal sample and who met the defined criteria for severe and critical disease. Suspected cases of COVID-19 with negative tests were excluded (Figure 1).

ICU - intensive care unit.

### Data collection

Patients’ electronic medical records were reviewed by trained physicians. Patient data included demographic characteristics, comorbidities, clinical data, vital signs, laboratory tests, type and length of time using organ support (mechanical ventilation, dialysis support, vasoactive drugs), and treatment measures (heparin, corticosteroids, antivirals, antibiotics, and immune therapy). The Simplified Acute Physiology Score 3 (SAPS 3) was performed upon ICU admission, and the Sequential Organ Failure Assessment (SOFA) was performed within the first three days in the ICU.^(17,18)^

The following laboratory tests were performed according to the ICU routine: high-sensitivity troponin T (hs-TnT), C-reactive protein (CRP), procalcitonin, D-dimer, liver enzymes, creatinine, creatine kinase (CK), total leukocytes, and platelet and lymphocyte counts. The radiologic assessment included chest radiography and computed tomography or chest angiotomography. Myocardial injury was defined as blood levels of cardiac troponin (cTn) above the 99th percentile upper reference limit (URL) on hs-TnT measured with the fifth generation Elecsys troponin T STAT assay (Roche, Basel, Switzerland) upon ICU admission. Levels > 14ng/L were considered elevated, regardless of new abnormalities on electrocardiography or echocardiography. Myocardial injury was considered acute if there was a rise and/or fall of cTn values, according to the Fourth Universal Definition of Myocardial Infarction (2018).^(19)^ Cardiovascular events considered were the composite of deep vein thrombosis (DVT), pulmonary embolism (PE), stroke, myocardial infarction (MI), acute limb ischemia (inferior or superior), mesenteric ischemia, heart failure (HF), and arrhythmia (supraventricular [atrial flutter, atrial fibrillation, atrial tachycardia], ventricular [ventricular tachycardia, ventricular fibrillation], or atrioventricular block).

The occurrence of cardiac complications during hospitalization was determined according to the European Society of Cardiology (ESC) diagnostic criteria for HF,^(20)^ acute coronary syndrome,^(21)^ DVT, and PE.^(22)^ The American College of Cardiology (ACC)/American Heart Association (AHA)/Heart Rhythm Society (HRS) 2006 key data elements and definitions for electrophysiological studies and procedures were used for arrythmias.^(23)^ We also used the ACC/AHA 2019 stroke guidelines^(24)^ and the European Society for Vascular Surgery (ESVS) 2020 limb ischemia guidelines.^(25)^ All patients were followed up until discharge or death. The 28-day mortality after COVID-19 was assessed.

### Statistical analysis

Categorical data are presented as absolute numbers and percentages, and continuous variables are presented as medians and interquartile ranges (25^th^ and 75^th^ percentiles). Continuous variables were compared using the nonparametric Mann-Whitney U test. Chi-square or Fisher’s exact tests were used to compare categorical variables.

Univariate and multivariate logistic regression models (enter elimination method) were used to determine predictors of myocardial injury in severe and critical patients admitted to the ICU because of COVID-19. The models were adjusted for age (≥ 60 years), sex (reference: male), body mass index (BMI; kg/m^2^), coexisting conditions (number of coexisting conditions; reference: no coexisting condition), arterial hypertension (AH), coronary artery disease (CAD), HF, asthma, chronic obstructive pulmonary disease (COPD), chronic kidney disease (CKD), cirrhosis, immune modulator use, diabetes, obesity (BMI: 30 to < 40kg/m^2^), morbid obesity (BMI ≥ 40kg/m^2^), and SOFA score (highest value within the first three days after ICU admission). The odds ratio (OR) and 95% confidence intervals (95%CI) were calculated for predictors.

Univariate and multivariate Cox proportional hazard models (enter elimination method) were used to determine predictors of cardiovascular events and 28-day mortality. The models were adjusted for demographics, coexisting conditions, risk score, laboratory tests (highest value within the first three days after ICU admission), glucocorticoid use, clinical outcomes, and cardiovascular events after COVID-19. Demographics included the following: age ≥ 60 years, sex (reference: male), body mass index (BMI; kg/m^2^), and baseline Chronic Kidney Disease Epidemiology Collaboration (CKD-EPI) < 60mL/min/1.73m^2^. Coexisting conditions included the following: number of coexisting conditions (reference: zero), AH, CAD, HF, asthma, COPD, CKD, cirrhosis, immune modulator use, diabetes, obesity (30 to < 40kg/m^2^), and morbid obesity (≥ 40kg/m^2^). The SOFA score was recorded as the highest value within the first three days after ICU admission. Glucocorticoid treatment included dexamethasone, methylprednisolone, and hydrocortisone. Clinical outcomes were as follows: acute kidney injury (AKI) based on kidney disease defined by the Kidney Disease: Improving Global Outcomes (KDIGO) criteria (within the first three days in the ICU), renal replacement therapy, mechanical ventilation, and bloodstream infection. Cardiovascular events included the following: number of cardiovascular events (no cardiovascular event, one cardiovascular event, ≥ 2 cardiovascular events; reference: no cardiovascular event), type of first cardiovascular event (no cardiovascular event, venous event, arterial event or HF and arrhythmia event; reference: no cardiovascular event), time from ICU admission to the cardiovascular event (days) (no cardiovascular event, early event [within the first week after ICU admission], late event [> 7 days after ICU admission]; reference: no cardiovascular event).

All variables included in the multivariate regression models were tested for multicollinearity using the variance inflation factor.

Patients who did not have any lab tests collected in the first three days after ICU admission were inferred to have had normal tests.

Cardiovascular events were divided into two groups: the early event group (occurring within the first week in the ICU) and the late event group (occurring after > 7 days in the ICU).

The purposeful selection process began with a univariate analysis of each of the variables above. Any variable having a univariate test with a p value < 0.10 was selected as a candidate for the multivariate analysis. Because of the large number of variables and to maintain the 1 in 10 rule (one predictive variable studied for every 10 events), after choosing the variables with p values < 0.10, we selected the highest values obtained in the Wald test to create the multivariate Cox proportional hazard model. The hazard ratio (HR) and 95%CI were calculated for predictors.

Cumulative survival graphics (Kaplan-Meier) were constructed to demonstrate differences in early event-free survival (all-cause 28-day mortality) according to myocardial injury development and the time to cardiovascular event (early or late).

The data were analyzed using the Statistical Package for Social Sciences (SPSS), version 26 (IBM Corporation, Armonk, NY). The p values < 0.05 were considered statistically significant (two-tailed).

## RESULTS

Of 1,200 consecutive patients with suspected COVID-19 admitted to the COVID-19 ICU, 567 patients with confirmed severe and critical forms of COVID-19 were included in the analysis (Figure 1). Table 1S (Supplementary material) presents the baseline characteristics of the included patients. The median age was 59 (48 - 71) years, and 324 patients (57.1%) were male. Among these patients, AH (56.1%) and diabetes (35.1%) were the most common coexisting conditions, and 7.9% and 4.1% had CAD and chronic HF, respectively (Table 1S - Supplementary material).

Over a period of 8 months, of the 567 patients with severe and critical forms of COVID-19, 273 patients (48.1%) had myocardial injury (Table 1S - Supplementary material). Of the 374 critical form patients (66%) with COVID-19, 235 patients (86.1%) had myocardial injury (Table 2S - Supplementary material). Patients with myocardial injury had higher SOFA and SAPS 3 scores on admission; 9 (6 - 12) *versus* 3 (3 - 7) and 65 (51 - 76) *versus* 44 (39 - 52), respectively. Compared with patients without myocardial injury, patients with myocardial injury were older (67 [56 - 76] years v *versus* 52 [42 - 63] years; p < 0.001). Moreover, coexisting conditions, including AH (71.8% *versus* 41.5%), diabetes (42.9% *versus* 27.9%), CAD (12.1% *versus* 4.1%), and CKD (9.5% *versus* 2.4%), were present more often among patients with myocardial injury (all p < 0.001) (Table 1S - Supplementary material).

In the assessment of laboratory results, patients with myocardial injury more frequently presented with procalcitonin > 2.0ng/mL (20.5% *versus* 3.7%, p < 0.001), lymphopenia (lymphocytes < 600 per mm^3^ (48.7% *versus* 29.9%, p < 0.001)), and D-dimer > 0.50µg/mL (97.4% *versus* 89.1%, p < 0.001) than patients without acute myocardial injury (Table 2S - Supplementary material). The prevailing course of the serum levels of CRP, procalcitonin, and D-dimer persisted elevated in the first 72 hours more frequently in the group with myocardial injury. At the same time, lymphocytes and platelets were lower and persisted at significantly lower counts than in patients without myocardial injury (Table 3S - Supplementary material).

Drugs used in the ICU, supportive therapy, and patient outcomes in both groups are shown in table 3S (Supplementary material). Patients with myocardial injury were mostly admitted to the ICU less than 12 hours after hospital admission (63.7% *versus* 46.9%), had more organ dysfunction on Days 1, 2, and 3, as reflected by higher SOFA scores, more frequently had renal failure upon ICU admission (54% *versus* 14.4%, p < 0.001), had a greater need for renal replacement therapy (30% *versus* 11.2%) and mechanical ventilation (85% *versus* 46.9%), and remained in the ICU longer (12 [6 - 20] days *versus* 9 [5 - 16] days) (Table 3S - Supplementary material).

**Figure 2:**
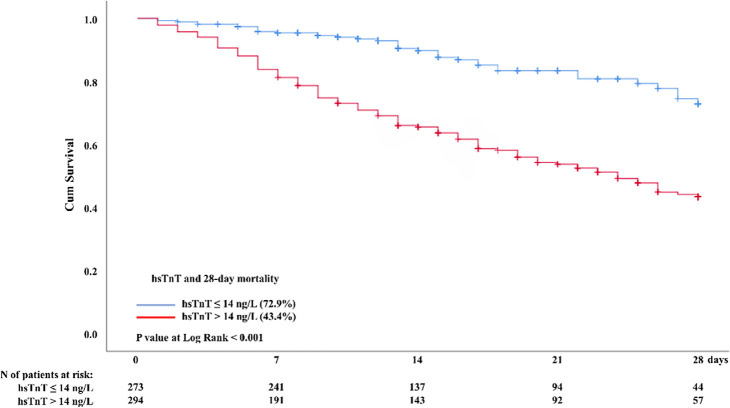
28-day mortality after COVID-19 for patients with or without myocardial injury.

The 28-day mortality was higher among patients with myocardial injury than among patients without myocardial injury (56.6% *versus* 27.1%, p < 0.001), as shown in table 1 and the Kaplan-Meier survival curves in figure 2.

**Table 1:** Multivariate logistic regression: independent predictors of myocardial injury in patients admitted to the intensive care unit due to COVID-19

	VIF^*^	Univariate analysis			Multivariate analysis		
		OR	95%CI	p value	OR	95%CI	p value
Age ≥ 60 years	1.52	5.02	3.51 - 7.17	0.000	3.10	2.06 - 4.67	0.000
Body mass index (kg/m^2^)	4.40	0.98	0.96 - 1.00	0.068			
Coexisting conditions (0; reference)	1.37	1.00	-				
Coexisting conditions (0 *versus* 1)	2.25	1.35	0.70 - 2.62	0.371			
Coexisting conditions (0 *versus* 2)	3.33	2.41	1.27 - 4.60	0.007			
Coexisting conditions (0 *versus* ≥ 3)	2.15	5.32	2.69 - 10.53	0.000			
Hypertension	1.11	3.59	2.53 - 5.10	0.000	2.36	1.56 - 3.59	0.000
Coronary artery disease	1.16	3.23	1.63 - 6.40	0.001			
Heart failure	1.23	4.08	1.49 - 11.15	0.006			
Chronic obstructive pulmonary disease	1.29	2.30	1.21 - 4.37	0.011			
Chronic kidney disease	1.11	4.32	1.84 - 10.12	0.001			
Immune modulators	1.32	2.76	0.85 - 8.90	0.090	4.60	1.20 - 17.74	0.026
Diabetes	2.73	1.94	1.37 - 2.75	0.000			
Obesity (30 to < 40kg/m^2^)	4.10	0.74	0.52 - 1.04	0.079			
SOFA score†	1.16	1.31	1.24 - 1.37	0.000	1.27	1.20 - 1.34	0.000

VIF - variance inflation factor; OR - odds ratio; 95%CI - 95% confidence interval; SOFA - Sequential Organ Failure Assessment.

* Variance inflation factor starts at 1 and has no upper limit; variance inflation factor exceeding 5 or 10 indicates high multicollinearity between this independent variable and the others; † the highest value within the first three days after intensive care unit admission.

hs-TnT - high-sensitivity troponin T.

Among the variables tested, age ≥ 60 years (OR 3.10; 95%CI 2.06 - 4.67), AH (OR 2.36; 95%CI 1.56 - 3.59), immune modulator use (OR 4.60; 95%CI 1.20 - 17.74), and SOFA score (OR 1.27; 95%CI 1.20 - 1.34) were independently associated with myocardial injury in the multivariate analysis.

Cardiovascular complications occurred in 113 of the 567 patients (19.9%) with severe and critical forms of COVID-19, with most events occurring in patients with myocardial injury (28.2% *versus* 12.2%, p < 0.001) and early (within the first week after ICU admission, 12%) *versus* late (more than seven days after ICU admission, 7.9%) (p < 0.001). Arrhythmias (6.5%), PE (6.3%), and DVT (4.2%) were the most frequent cardiovascular events. Venous thrombotic events (8.8%) were the most frequently observed first cardiovascular event, compared with HF or arrhythmia (7.1%) and arterial events (4.1%) (Table 4S - Supplementary material).

**Figure 3:**
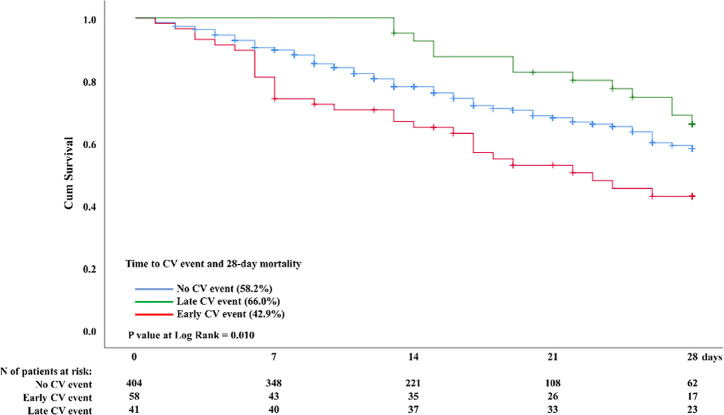
28-day mortality after COVID-19 according to the time of cardiovascular events.

For patients with cardiovascular complications, the 28-day mortality after COVID-19 was higher among those who had early events than among those who had late events, 57.1% *versus* 34%, respectively, as shown in the Kaplan - Meier survival curves in figure 3. The multivariate analysis by Cox proportional hazards models showed the following variables as independent predictors of cardiovascular events: age ≥ 60 years (HR 1.97; 95%CI 1.30 - 3.00), lymphopenia (lymphocytes < 600 per mm^3^; HR 1.55; 95%CI 1.04 - 2.31), CK > 308U/L (HR 1.97; 95%CI 1.31 - 2.97), and hydrocortisone use (HR 2.56; 95%CI 1.71 - 3.84) (Table 2).

CV - cardiovascular.

**Table 2:** Multivariate analysis by Cox proportional hazards models: independent predictors of cardiovascular event^*^ in patients admitted to the intensive care unit due to COVID-19†

	VIF‡	Univariate analysis			Multivariate analysis		
		OR	95%CI	p value	OR	95%CI	p value
Age ≥ 60 years	1.28	2.36	1.56 - 3.56	0.000	1.97	1.30 - 3.00	0.001
SOFA score (highest)	1.58	1.09	1.04 - 1.15	0.000			
Lymphocytes (< 600 per mm^3^)	1.05	1.75	1.18 - 2.61	0.006	1.55	1.04 - 2.31	0.030
Creatine kinase (>308U/L)	1.11	1.92	1.28 - 2.89	0.002	1.97	1.31 - 2.97	0.001
High sensitivity troponin T (>14ng/L)	1.68	2.08	1.37 - 3.14	0.001			
CKD-EPI < 60mL/min/1.73 m^2^ on ICU admission	1.43	1.62	1.08 - 2.44	0.020			
Dexamethasone	1.10	0.71	0.47 - 1.07	0.098			
Hydrocortisone	1.13	2.83	1.90 - 4.22	0.000	2.56	1.71 - 3.84	0.000

VIF - variance inflation factor; OR - odds ratio; 95% CI - 95% confidence interval; SOFA - Sequential Organ Failure Assessment; CKD-EPI - Chronic Kidney Disease Epidemiology Collaboration.

* Cardiovascular event: composite of deep vein thrombosis, pulmonary embolism, stroke, myocardial infarction, acute limb ischemia (inferior or superior), mesenteric ischemia, heart failure, and arrhythmia (supraventricular [atrial flutter, atrial fibrillation, atrial tachycardia], ventricular [ventricular tachycardia, ventricular fibrillation], or atrioventricular block); † palliative care patients were excluded from this analysis; ‡ variance inflation factor starts at 1 and has no upper limit; variance inflation factor exceeding 5 or 10 indicates high multicollinearity between this independent variable and the others.

## DISCUSSION

The main findings of our study in severe and critical forms of COVID-19 patients admitted to the ICU were as follows: myocardial injury was persistent and was associated with illness severity and with a more intense inflammatory response and worse outcomes; advanced age, AH, immune modulator use, and a high SOFA score were independent predictors of myocardial injury; cardiovascular events, particularly thrombotic events, HF, and cardiac arrhythmias, more than doubled in patients with myocardial injury and were independently associated with age, lymphocytopenia, higher CK levels, and hydrocortisone use; and the occurrence of an early cardiovascular event during the ICU stay was associated with higher mortality compared with late or no events.

Myocardial injury was diagnosed in almost half of the severe and critical COVID-19 patients admitted to our ICU and was associated with disease severity at admission. A more intense inflammatory response ensued in the next three days, with more organ dysfunction, a greater need for mechanical ventilator support, and higher 28-day mortality. Indeed, patients with myocardial injury had a shorter time between hospital and ICU admission (less than 12 hours), reflecting a more severe presentation at hospital admission.

The largest available study of myocardial injury is a multicenter, retrospective analysis of 2,736 hospitalized patients, of whom 36% had evidence of myocardial injury at the time of presentation.^(12)^ Twenty-eight-day mortality was markedly higher in patients with elevated TnT levels than in patients with normal TnT levels in our study (56% *versus* 18.4%), and other authors have reported a fourfold increase in the risk of death.^(26-30)^ Therefore, elevated troponin might be used to identify patients at high risk of a complicated course and death.

This report shows underlying AH, older age, immune modulator use, and a higher SOFA score as independent predictors of myocardial injury. Preexisting CAD and AH were more prevalent in patients with cardiac injury than in those without myocardial injury. According to other authors,^(31,32)^ elderly patients with underlying diseases are more likely to be infected with SARS-CoV-2 and tend to be severely ill, especially those with AH, CAD, and diabetes. In addition, age over 60 years increased the odds of myocardial injury more than three times, and the use of immune modulators was also related to myocardial injury. An explanation for these observations is that both factors are possibly related to a more aggressive viral infection in these conditions.

In our study, cardiovascular complications were divided into venous or arterial events, arrhythmias, or HF. Arrhythmias affected 6.7% of patients, followed by PE in 6.5% and DVT in 4.2%, among others, for a total of 18.7% of patients having venous events. Arterial events occurred in 0.7% of patients, and HF occurred in 1.8% of patients. In our study, HF including “probable myocarditis” was present in 2.0% of patients. Indeed, in other studies, the data on myocarditis in COVID-19 are sparse, with the reported prevalence ranging up to 12%.^(33-35)^ VTE is increasingly being recognized in COVID-19 patients and is often due to underlying coagulopathy. Grillet et al.^(36)^ recently reported the prevalence of acute PE in 23% of severe COVID-19 cases. We had only 34 cases (6.8%) of PE, which may be underestimated due to the difficulty in diagnosing critically ill COVID-19 patients, since transport for imaging tests poses risks of severe hypoxemia and/or hemodynamic instability.

Age ≥ 60 years was an independent predictor of cardiovascular complications, with twice the risk over younger patients. Other merging independent predictors in the multivariate analysis were lymphopenia, CK > 308U/L, and hydrocortisone use (which more than doubled the risk). Studies suggest that participants with overt or subacute inflammatory diseases have an elevated risk of atherothrombotic disease and HF.^(37)^ These mechanisms might be overwhelmingly accelerated in critical COVID-19 cases.

The 28-day mortality after COVID-19 was higher in patients with early cardiovascular complications (within seven days after ICU admission). The mechanism by which this happens is unclear but may be related to cardiovascular event effects at a more critical phase further complicating the course of COVID-19. Attention to early diagnosis and treatment of cardiovascular complications in patients with myocardial injury might be of foremost importance to achieve better results.

Our study has some limitations. This study has a retrospective design and lacks some specific information about cardiovascular complications, such as echocardiography, magnetic resonance, and computed tomography, which were not performed due to transport-associated risks of performing the exams. Conversely, the study’s strengths are the large number of patients receiving standard-of-care treatment. Forty-seven patients (9.7%) had no cardiac troponin samples within the first three days of admission, but for regression analysis purposes, these patients were considered to have no myocardial injury. Despite representing a loss of almost 10% of patients, the presence of myocardial injury continued to show an association with relevant clinical events, corroborating the strength of this risk marker for COVID-19 patients. We were unable to include the clinical severity and mortality of patients with different biological behavior of the COVID-19 variants of concern found in our region because the genetic mapping of our study patients was not conducted for the different variants.

## CONCLUSION

In conclusion, it is evident that myocardial injury is a frequently encountered complication in severe and critical intensive care unit patients with COVID-19 and that advanced age, arterial hypertension, the use of immunomodulators and high Sequential Organ Failure Assessment score are independent predictors for the occurrence of COVID-19 and for greater severity of the disease. Cardiovascular complications are also common in these patients and lead to higher mortality. However, the exact mechanism regarding cardiovascular involvement remains unclear. In our study, among patients who had cardiovascular complications, the highest 28-day mortality occurred in patients who had the cardiovascular event within the first seven days of intensive care unit admission. In addition to preventing SARS-CoV-2 infections, it is essential to prevent cardiovascular involvement to decrease the morbidity and mortality of these patients.

## References

[1] World Health Organization (WHO) Weekly Epidemiological.

[2] Zaim S, Chong JH, Sankaranarayanan V, Harky A (2020). COVID-19 and multiorgan response. Curr Probl Cardiol.

[3] Cawcutt K, Kalil AC (2017). Pneumonia with bacterial and viral coinfection. Curr Opin Crit Care.

[4] Zhou F, Yu T, Du R, Fan G, Liu Y, Liu Z (2020). Clinical course and risk factors for mortality of adult
inpatients with COVID-19 in Wuhan, China: a retrospective cohort
study. Lancet.

[5] Huang C, Wang Y, Li X, Ren L, Zhao J, Hu Y (2020). Clinical features of patients infected with 2019 novel
coronavirus in Wuhan, China. Lancet.

[6] Centers for Disease Control and Prevention (CDC) (2022). What you need to know about variants [vídeo]. CDC.

[7] Guo T, Fan Y, Chen M, Wu X, Zhang L, He T (2020). Cardiovascular implications of fatal outcomes of patients with
coronavirus disease 2019 (COVID-19). JAMA Cardiol.

[8] Shi S, Qin M, Shen B, Cai Y, Liu T, Yang F (2020). Association of cardiac injury with mortality in hospitalized
patients with COVID-19 in Wuhan, China. JAMA Cardiol.

[9] Chen T, Wu D, Chen H, Yan W, Yang D, Chen G (2020). Clinical characteristics of 113 deceased patients with
coronavirus disease 2019: retrospective study. BMJ.

[10] Smeeth L, Thomas SL, Hall AJ, Hubbard R, Farrington P, Vallance P (2004). Risk of myocardial infarction and stroke after acute infection or
vaccination. N Engl J Med.

[11] Wang D, Hu B, Hu C, Zhu F, Liu X, Zhang J (2020). Clinical characteristics of 138 hospitalized patients with 2019
novel coronavirus-infected pneumonia in Wuhan, China. JAMA.

[12] Lala A, Jonhson KW, Januzzi JL, Russak AJ, Paranjpe I, Richter F, Zhao S, Somani S, Van Vleck T, Vaid A, Chaudhry F, De Freitas JK, Fayad ZA, Pinney SP, Levin M, Charney A, Bagiella E, Narula J, Glicksberg BS, Nadkarni G, Mancini DM, Fuster V, Mount Sinai COVID (2020). Informatics Center. Prevalence and impact of myocardial injury in
patients hospitalized with COVID-19 infection. J Am Coll Cardiol.

[13] Richardson S, Hirsch JS, Narasimhan M, Crawford JM, McGinn T, Davidson KW, the Northwell COVID-19 Research Consorti (2020). Presenting characteristics, comorbidities, and outcomes among
5700 patients hospitalized with COVID-19 in the New York City
area. JAMA.

[14] Bavishi C, Bonow RO, Trivedi V, Abbott JD, Messerli FH, Bhatt DL (2020). Special Article - Acute myocardial injury in patients
hospitalized with COVID-19 infection: a review. Prog Cardiovasc Dis.

[15] Basso C, Leone O, Rizzo S, De Gaspari M, van der Wal AC, Aubry MC (2020). Pathological features of COVID-19-associated myocardial injury: a
multicentre cardiovascular pathology study. Eur Heart J.

[16] Alhazzani W, Moller MH, Arabi YM, Loeb M, Gong MN, Fan E (2020). Surviving Sepsis Campaign: Guidelines on the Management of
Critically Ill Adults with Coronavirus Disease 2019
(COVID-19). Crit Care Med.

[17] Zimmerman JE, Wagner DP, Draper EA, Wright L, Alzola C, Knaus WA (1998). Evaluation of acute physiology and chronic health evaluation III
predictions of hospital mortality in an independent database. Crit Care Med.

[18] Vicent JL, Moreno R, Takala J, Willatts S, De Mendonça A, Bruining H (1996). The SOFA (Sepsis-related Organ Failure Assessment) score to
describe organ dysfunction/failure. On behalf of the Working Group on
Sepsis-Related Problems of the European Society of Intensive Care
Medicine. Intensive Care Med.

[19] Thygesen K, Alpert JS, Jaffe AS, Chaitman BR, Bax JJ, Morrow DA, White HD, Executive Group on behalf of the Joint European Society of
Cardiology (ESC)/American College of Cardiology (ACC)/American Heart
Association (AHA)/World Heart Federation (WHF) Task Force for the
Universal Definition of Myocardial Infarction (2018). Fourth Universal Definition of Myocardial Infarction
(2018). J Am Coll Cardiol.

[20] Ponikowski P, Voors AA, Anker SD, Bueno H, Cleland JG, Coats AJ, Falk V, González-Juanatey JR, Harjola VP, Jankowska EA, Jessup M, Linde C, Nihoyannopoulos P, Parissis JT, Pieske B, Riley JP, Rosano GM, Ruilope LM, Ruschitzka F, Rutten FH, van der Meer P, ESC Scientific Document Group (2016). 2016 ESC Guidelines for the diagnosis and treatment of acute and
chronic heart failure: The Task Force for the diagnosis and treatment of
acute and chronic heart failure of the European Society of Cardiology (ESC).
Developed with the special contribution of the Heart Failure Association
(HFA) of the ESC. Eur Heart J.

[21] Roffi M, Patrono C, Collet JP, Mueller C, Valgimigli M, Andreotti F, Bax JJ, Borger MA, Brotons C, Chew DP, Gencer B, Hasenfuss G, Kjeldsen K, Lancellotti P, Landmesser U, Mehilli J, Mukherjee D, Storey RF, Windecker S, ESC Scientific Document Group (2016). 2015 ESC Guidelines for the management of acute coronary
syndromes in patients presenting without persistent ST-segment elevation:
Task Force for the Management of Acute Coronary Syndromes in Patients
Presenting without Persistent ST-Segment Elevation of the European Society
of Cardiology (ESC). Eur Heart J.

[22] Konstantinides SV, Meyer G, Becattini C, Bueno H, Geersing GJ, Harjola VP, Huisman MV, Humbert M, Jennings CS, Jiménez D, Kucher N, Lang IM, Lankeit M, Lorusso R, Mazzolai L, Meneveau N, Ní Áinle F, Prandoni P, Pruszczyk P, Righini M, Torbicki A, Van Belle E, Zamorano JL, ESC Scientific Document Group (2020). 2019 ESC Guidelines for the diagnosis and management of acute
pulmonary embolism developed in collaboration with the European Respiratory
Society (ERS). Eur Heart J.

[23] Buxton AE, Calkins H, Callans DJ, DiMarco JP, Fisher JD, Greene HL, Haines DE, Hayes DL, Heidenreich PA, Miller JM, Poppas A, Prystowsky EN, Schoenfeld MH, Zimetbaum PJ, Heidenreich PA, Goff DC, Grover FL, Malenka DJ, Peterson ED, Radford MJ, Redberg RF, American College of Cardiology, American Heart Association Task Force on Clinical Data
Standards, ACC/AHA/HRS Writing Committee to Develop Data Standards on
Electrophysiology (2006). ACC/AHA/HRS 2006 key data elements and definitions for
electrophysiological studies and procedures: a report of the American
College of Cardiology/American Heart Association Task Force on Clinical Data
Standards (ACC/AHA/HRS Writing Committee to Develop Data Standards on
Electrophysiology). J Am Coll Cardiol.

[24] Powers WJ, Rabinstein AA, Ackerson T, Adeoye OM, Bambakidis NC, Becker K (2019). Guidelines for the Early Management of Patients With Acute
Ischemic Stroke: 2019 Update to the 2018 Guidelines for the Early Management
of Acute Ischemic Stroke: A Guideline for Healthcare Professionals From the
American Heart Association/American Stroke Association. Stroke.

[25] Bjorck M, Earnshaw JJ, Acosta S, Bastos Gonçalves F, Cochennec F, Debus ES (2020). Editor’s Choice - European Society for Vascular Surgery (ESVS)
2020 Clinical Practice Guidelines on the Management of Acute Limb
Ischaemia. Eur J Vasc Endovasc Surg.

[26] Manocha KK, Kirzner J, Ying X, Yeo I, Peltzer B, Ang B (2021). Troponin and other biomarker levels and outcomes among patients
hospitalized with COVID-19: derivation and validation of the HA2T2 COVID-19
mortality risk score. J Am Heart Assoc.

[27] Zou F, Qian Z, Wang Y, Zhao Y, Bai J (2020). Cardiac injury and COVID-19: a systematic review and
meta-analysis. CJC Open.

[28] Almeida Junior GL, Braga F, Jorge JK, Nobre GF, Kalichsztein M, Faria PM (2020). Prognostic value of troponin-T and B-type natriuretic peptide in
patients hospitalized for COVID-19. Arq Bras Cardiol.

[29] Kavsak PA, Hammarsten O, Worster A, Smith SW, Apple FS (2021). Cardiac troponin testing in patients with COVID-19: a strategy
for testing and reporting results. Clin Chem.

[30] Cordeanu EM, Duthil N, Severac F, Lambach H, Tousch J, Jambert L (2020). Prognostic value of troponin elevation in COVID-19 hospitalized
patients. J Clin Med.

[31] National Health Commission & State Administration of Traditional
Chinese Medicine Diagnosis and Treatment Protocol for Novel Coronavirus Pneumonia (Trial
Version 7).

[32] COVID-ICU Group on behalf of the REVA Network and the COVID-ICU
Investigators (2021). Clinical characteristics and day-90 outcomes of 4244 critically
ill adults with COVID-19: a prospective cohort study. Intensive Care Med.

[33] Deng Q, Hu B, Zhang Y, Wang H, Zhou X, Hu W (2020). Suspected myocardial injury in patients with COVID-19: evidence
from front-line clinical observation in Wuhan, China. Int J Cardiol.

[34] Inciardi RM, Lupi L, Zaccone G, Italia L, Raffo M, Tomasoni D (2020). Cardiac involvement in a patient with coronavirus disease 2019
(COVID-19). JAMA Cardiol.

[35] Doyen D, Moceri P, Ducreux D, Dellamonica J (2020). Myocarditis in a patient with COVID-19: a cause of raised
troponin and ECG changes. Lancet.

[36] Grillet F, Behr J, Calame P, Aubry S, Delabrousse E (2020). Acute pulmonary embolism associated with COVID-19 pneumonia
detected with pulmonary CT angiography. Radiology.

[37] Zidar DA, Al-Kindi SG, Liu Y, Krieger NI, Perzynski AT, Osnard M (2019). Association of lymphopenia with risk of mortality among adults in
the US general population. JAMA Netw Open.

