# Supplementary material for: Myocardial injury and cardiovascular complications in COVID-19: a cohort study in severe and critical patients
Source: Rev Bras Ter Intensiva. 2022 Oct-Dec;34(4):443–51. doi: 10.5935/0103-507X.20220440-en (PMC9986998; doi:10.5935/0103-507X.20220440-en)
Supplement: Supplementary file 1 [file rbti-34-04-0443-suppl1.pdf]

Myocardial injury and cardiovascular complications in COVID-19: a cohort study in severe and critical patients

Lesão miocárdica e complicações cardiovasculares na COVID-19: estudo de coorte em pacientes graves e críticos

Ana Palmira L. Neves[1], Mauricio Nassau Machado[2], Joelma Vilafanha Gandolfi[1], Luana Fernandes Machado[1], Juliana Devós Syrio[1], Graziella Luckmeyer[1], Suzana Margareth Lobo[1]

Table 1S - Clinical characteristics and coexisting conditions of patients with COVID-19 according to the development of myocardial injury

	Overall n = 567	hs-TnT ≤ 14ng/L n = 294	hs-TnT > 14ng/L n = 273	p value
Critical COVID-19	374 (66.0)	139 (47.3)	235 (86.1)	< 0.001
Demographics				
Age (years)	59 (48 - 71)	52 (42 - 63)	67 (56 - 76)	< 0.001
≥ 60	280 (49.4)	91 (31.0)	189 (69.2)	< 0.001
18 to < 40	68 (12.0)	56 (19.0)	12 (4.4)	< 0.001
40 to < 60	218 (38.5)	147 (50.0)	71 (26.1)	< 0.001
60 to < 80	222 (39.2)	79 (26.9)	143 (52.6)	< 0.001
≥ 80	58 (10.2)	12 (4.1)	46 (16.9)	< 0.001
Male gender	324 (57.1)	174 (59.2)	150 (54.9)	0.308
Severity score at admission				
SOFA in ICU admission	4 (3 - 9)	3 (2 - 5)	7 (4 - 11)	< 0.001
SAPS 3	51 (42 - 69)	44 (39 - 52)	65 (51 - 76)	< 0.001
Symptoms to ICU admission. days	7 (5 - 10)	8 (6 - 10)	7 (5 - 10)	0.006
Hospital admission to ICU admission				
< 12 hours	312 (55.0)	138 (46.9)	174 (63.7)	< 0.001
12 to < 24 hours	95 (16.8)	55 (18.7)	40 (14.7)	0.196
24 to < 48 hours	75 (13.2)	49 (16.7)	26 (9.5)	0.012
2 to < 5 days	50 (8.8)	33 (11.2)	17 (6.2)	0.036
≥ 5 days	35 (6.2)	19 (6.5)	16 (5.9)	0.766
Coexisting conditions (#)	512 (90.3)	255 (86.7)	257 (94.1)	0.003
1	171 (30.2)	110 (37.4)	61 (22.3)	< 0.001
2	201 (35.4)	101 (34.4)	100 (36.6)	0.571
≥ 3	140 (24.7)	44 (15.0)	96 (35.2)	< 0.001
Cardiovascular disease	326 (57.5)	124 (42.2)	202 (74.0)	< 0.001
Hypertension	318 (56.1)	122 (41.5)	196 (71.8)	< 0.001
Coronary artery disease	45 (7.9)	12 (4.1)	33 (12.1)	< 0.001
Heart failure	23 (4.1)	5 (1.7)	18 (6.6)	0.003
Chronic disease	62 (10.9)	25 (8.5)	37 (13.6)	0.054
Asthma	17 (3.0)	10 (3.4)	7 (2.6)	0.559
Chronic obstructive pulmonary disease	45 (7.9)	15 (5.1)	30 (11.0)	0.010
Chronic kidney disease	33 (5.8)	7 (2.4)	26 (9.5)	< 0.001
Cirrhosis	5 (0.9)	4 (1.4)	1 (0.4)	0.375
Immunosuppression	16 (2.8)	5 (1.7)	11 (4.0)	0.094
HIV infection	2 (0.4)	1 (0.3)	1 (0.4)	1.000
Immune modulators	14 (2.5)	4 (1.4)	10 (3.7)	0.078
Metabolic disease	358 (63.1)	187 (63.6)	171 (62.6)	0.811
Diabetes	199 (35.1)	82 (27.9)	117 (42.9)	< 0.001
Obesity (30 to < 40kg/m²)	210 (37.4)	119 (40.9)	91 (33.7)	0.079
Morbid obesity (≥ 40kg/m²)	51 (9.1)	28 (9.6)	23 (8.5)	0.650
Other	19 (3.4)	12 (4.1)	7 (2.6)	0.316
Pregnancy	8 (1.4)	8 (2.7)	0 (0.0)	0.008

hs-TnT - high-sensitivity troponin T; SOFA - Sequential Organ Failure Assessment; ICU - intensive care unit; SAPS 3 - Simplified Acute Physiology Score 3. Results expressed as n (%) or medians and interquartile ranges (25th and 75th percentiles).

Table 2S - Risk scores and laboratory tests of patients with COVID-19 according to the development of myocardial injury

	Overall n = 567	hs-TnT ≤ 14ng/L n = 294	hs-TnT > 14ng/L n = 273	p value
C-reactive protein (mg/dL; normal range 0.00 - 0.50)				
Day 1	13.73 (7.60 - 22.93)	11.12 (6.49 - 20.67)	15.90 (9.59 - 24.17)	< 0.001
Day 2	13.18 (7.22 - 23.37)	11.10 (5.79 - 20.17)	16.01 (9.01 - 27.52)	< 0.001
Day 3	10.96 (5.60 - 20.64)	8.34 (4.32 - 17.87)	12.97 (6.68 - 22.82)	< 0.001
C-reactive protein (highest)	17.50 (10.29 - 27.28)	15.13 (7.97 - 24.57)	20.62 (13.26 - 30.18)	< 0.001
Procalcitonin (ng/mL)				
Day 1	0.2 (0.1 - 0.6)	0.1 (0.1 - 0.2)	0.4 (0.2 - 0.9)	< 0.001
Day 2	0.2 (0.1 - 0.7)	0.1 (0.1 - 0.3)	0.5 (0.2 - 1.4)	< 0.001
Day 3	0.2 (0.1 - 1.0)	0.2 (0.1 - 0.4)	0.5 (0.2 - 1.7)	< 0.001
Procalcitonin (highest)	0.3 (0.1 - 0.9)	0.2 (0.1 - 0.3)	0.6 (0.2 - 1.9)	< 0.001
Procalcitonin (low risk severe sepse < 0.5/high risk severe sepse > 2.0)				
Procalcitonin (< 0.5ng/mL)	319 (56.3)	208 (70.7)	111 (40.7)	< 0.001
Procalcitonin (>2.0ng/mL)	67 (11.8)	11 (3.7)	56 (20.5)	< 0.001
Lymphocytes (per mm³; normal range 600 to 3960)				
Day 1	820 (580 - 1185)	880 (653 - 1250)	750 (504 - 1080)	< 0.001
Day 2	890 (602 - 1323)	970 (684 - 1325)	830 (565 - 1325)	0.013
Day 3	920 (625 - 1320)	978 (698 - 1415)	840 (520 - 1210)	< 0.001
Lymphocytes (lowest)	680 (480 - 940)	760 (550 - 1010)	609 (425 - 855)	< 0.001
Lymphocytes (highest)	1135 (810 - 1593)	1203 (878 - 1634)	1040 (740 - 1525)	0.010
Lymphocytes (< 600 per mm³)	221 (39.0)	88 (29.9)	133 (48.7)	< 0.001
Lymphocytes (> 3,960 per mm³)	3 (0.5)	0 (0.0)	3 (1.1)	0.111
Platelets (×10³ per mm³; normal range 150 - 450)				
Day 1	208 (155 - 273)	215 (166 - 274)	201 (146 - 270)	0.012
Day 2	228 (170 - 285)	236 (179 - 298)	218 (160 - 280)	0.019
Day 3	236 (187 - 307)	262 (195 - 318)	223 (177 - 293)	0.001
Platelets (lowest)	199 (149 - 252)	204 (158 - 259)	186 (134 - 248)	0.004
Platelets (highest)	253 (197 - 321)	273 (213 - 329)	239 (189 - 307)	0.001
Platelets (< 150 x10³ per mm³)	143 (25.2)	59 (20.1)	84 (30.8)	0.003
Platelets (> 450 x10³ per mm³)	26 (4.6)	17 (5.8)	9 (3.3)	0.157
Lactate dehydrogenase (U/L; normal range 1 - 250)				
Day 1	462 (365 - 603)	434 (345 - 530)	504 (377 - 678)	< 0.001
Day 2	464 (369 - 595)	443 (354 - 534)	499 (381 - 645)	0.002
Day 3	434 (351 - 559)	428 (349 - 539)	442 (352 - 582)	0.176
Lactate dehydrogenase (lowest)	415 (327 - 531)	388 (316 - 476)	445 (340 - 570)	< 0.001
Lactate dehydrogenase (highest)	497 (394 - 655)	473 (388 - 603)	550 (423 - 695)	< 0.001
Lactate dehydrogenase (> 250U/L)	541 (95.4)	277 (94.2)	264 (96.7)	0.157
D-dimer (μg/mL; normal range < 0.50)				
Day 1	1.33 (0.81 - 2.67)	1.00 (0.63 - 1.62)	1.88 (1.05 - 3.72)	< 0.001
Day 2	1.61 (0.86 - 3.43)	1.13 (0.64 - 2.30)	2.35 (1.26 - 4.15)	< 0.001
Day 3	1.59 (0.92 - 2.87)	1.26 (0.74 - 1.91)	2.11 (1.25 - 3.91)	< 0.001
D-dimer (lowest)	1.15 (0.70 - 2.17)	0.85 (0.55 - 1.44)	1.59 (0.95 - 2.94)	< 0.001
D-dimer (highest)	1.87 (1.07 - 3.81)	1.43 (0.86 - 2.56)	2.65 (1.45 - 5.48)	< 0.001
D-dimer (> 0.50μg/mL)	528 (93.1)	262 (89.1)	266 (97.4)	< 0.001
High sensitivity troponin T (ng/L; 99th percentile upper reference limit [14ng/L])				
Day 1	12 (7 - 35)	7 (5 - 9)	31 (15 - 81)	< 0.001
Day 2	13 (7 - 51)	7 (5 - 9)	42 (18 - 123)	< 0.001
Day 3	18 (8 - 70)	7 (5 - 10)	54 (21 - 158)	< 0.001
# High sensitivity troponin T samples	2 (1 - 2)	2 (1 - 2)	2 (1 - 3)	< 0.001
High sensitivity troponin T (lowest)	11 (7 - 32)	6 (4 - 9)	28 (15 - 70)	< 0.001
High sensitivity troponin T (highest)	17 (8 - 60)	8 (5 - 10)	52 (24 - 143)	< 0.001
High sensitivity troponin T (> 14ng/L)	273 (48.1)	0 (0.0)	273 (100.0)	-
High sensitivity troponin T (delta; ng/L)	1 (0 - 7)	0 (0 - 2)	8 (0 - 52)	< 0.001
Serum creatinine (mg/dL; normal range 0.7 to 1.2)				
Day 1	1.0 (0.7 - 1.4)	0.8 (0.7 - 1.1)	1.3 (0.9 - 2.0)	< 0.001
Day 2	1.0 (0.7 - 1.5)	0.8 (0.7 - 1.0)	1.3 (0.9 - 2.2)	< 0.001
Day 3	1.0 (0.7 - 1.8)	0.8 (0.6 - 1.1)	1.5 (1.0 - 2.7)	< 0.001
Serum creatinine (highest)	1.2 (0.8 - 2.0)	0.9 (0.8 - 1.3)	1.7 (1.2 - 3.1)	< 0.001
CKD-EPI on ICU admission (mL/min/1.73 m²)	77 (45 - 101)	95 (74 - 108)	56 (29 - 81)	< 0.001
CKD-EPI < 60mL/min/1.73 m² on ICU admission	189 (33.5)	42 (14.4)	147 (54.0)	< 0.001

hs-TnT - high-sensitivity troponin T; CKD-EPI - Chronic Kidney Disease Epidemiology Collaboration; ICU - intensive care unit. *A rising pattern (delta hs-cTnT) was considered to be an absolute increase of >7ng/L above the 99th percentile if the first value was ≤ 14ng/L to align with reports from others and the European Society of Cardiology guidance.[1] If the first value was >14ng/L, an increase > 7ng/L of the first measurement was considered as a significant delta change.[1] Results expressed as medians and interquartile ranges (25th and 75th percentiles) or n (%).

1. Adapted from: Machado MN, Rodrigues FB, Nakazone MA, Martin DF, Sabbag AT, Grigolo IH, et al. Prediction of death after noncardiac surgery: potential advantage of using high-sensitivity troponin T as a continuous variable. J Am Heart Assoc. 2021 Mar 16;10(6):e018008.

Table 3S - Therapeutics, organ dysfunction, type of organ support and outcomes of patients with COVID-19 according to the development of myocardial injury

	Overall<br>n = 567	hs-TnT ≤ 14ng/L<br>n = 294	hs-TnT > 14ng/L<br>n = 273	p value
Supportive therapy and drugs				
Azithromycin	492 (86.8)	249 (84.7)	243 (89.0)	0.129
Hydroxychloroquine	6 (1.1)	5 (1.7)	1 (0.4)	0.218
Glucocorticoid	498 (87.8)	264 (89.8)	234 (85.7)	0.137
Dexamethasone	382 (67.4)	228 (77.6)	154 (56.4)	< 0.001
Methylprednisolone	272 (48.0)	140 (47.6)	132 (48.4)	0.861
Hydrocortisone	143 (25.2)	53 (18.0)	90 (33.0)	< 0.001
Experimental drug (clinical trial medicine; yes/no)	114 (20.1)	86 (29.3)	28 (10.3)	< 0.001
Tocilizumab	38 (6.7)	29 (9.9)	9 (3.3)	0.002
Heparin dose regimen*				
Heparin (prophylactic dose)	455 (80.2)	259 (88.1)	196 (71.8)	< 0.001
Heparin (intermediate dose)	338 (59.6)	171 (58.2)	167 (61.2)	0.466
Heparin (full dose)	202 (35.6)	82 (27.9)	120 (44.0)	< 0.001
Unfractionated heparin (full dose)	77 (13.6)	17 (5.8)	60 (22.0)	< 0.001
Low molecular weight heparin (full dose)	148 (26.1)	70 (23.8)	78 (28.6)	0.197
Days on full dose heparin	0 (0 - 3)	0 (0 - 1)	0 (0 - 5)	< 0.001
Norepinephrine (yes/no)	361 (63.7)	131 (44.6)	230 (84.2)	< 0.001
Norepinephrine (days)	3 (0 - 8)	0 (0 - 6)	5 (2 - 10)	< 0.001
Norepinephrine dose (highest; μg/kg/minute)	0.10 (0.00 - 0.70)	0.00 (0.00 - 0.20)	0.35 (0.09 - 1.25)	< 0.001
Vasopressin (yes/no)	126 (22.2)	38 (12.9)	88 (32.2)	< 0.001
Vasopressin (days)	0 (0 - 0)	0 (0 - 0)	0 (0 - 1)	< 0.001
Dobutamine (yes/no)	37 (6.5)	7 (2.4)	30 (11.0)	< 0.001
Dobutamine (days)	0 (0 - 0)	0 (0 - 0)	0 (0 - 0)	< 0.001
Mechanical ventilation	370 (65.3)	138 (46.9)	232 (85.0)	< 0.001
Renal replacement therapy	115 (20.3)	33 (11.2)	82 (30.0)	< 0.001
Outcomes				
Organ dysfunction				
SOFA Day 1	4 (3 - 9)	3 (2 - 5)	7 (4 - 11)	< 0.001
SOFA Day 2	4 (3 - 9)	3 (2 - 5)	8 (4 - 11)	
SOFA Day 3	5 (3 - 9)	3 (2 - 6)	8 (5 - 11)	
SOFA score (highest)†	6 (3 - 10)	3 (3 - 7)	9 (6 - 12)	
Increase in SCr ≥ 0.3mg/dL	189 (33.3)	57 (19.4)	132 (48.4)	< 0.001
Increase in SCr ≥ 50%	123 (21.7)	38 (12.9)	85 (31.1)	< 0.001
AKI based on KDIGO criteria (first three days in ICU)	193 (34.0)	60 (20.4)	133 (48.7)	< 0.001
Hospital admission to ICU admission				
< 12 hours	312 (55.0)	138 (46.9)	174 (63.7)	< 0.001
12 to < 24 hours	95 (16.8)	55 (18.7)	40 (14.7)	0.196
24 to < 48 hours	75 (13.2)	49 (16.7)	26 (9.5)	0.012
2 to < 5 days	50 (8.8)	33 (11.2)	17 (6.2)	0.036
≥ 5 days	35 (6.2)	19 (6.5)	16 (5.9)	0.766
ICU length of stay	11 (5 - 18)	9 (5 - 16)	12 (6 - 20)	0.002
Hospital length of stay	15 (9 - 24)	14 (9 - 21)	16 (8 - 26)	0.493
Hospital mortality	242 (42.7)	72 (24.5)	170 (62.3)	< 0.001
Hospital length of stay up to 28 days	15 (9 - 24)	14 (9 - 21)	16 (8 - 26)	0.487
28-day mortality	207 (36.5)	54 (18.4)	153 (56.0)	< 0.001
Palliative care	64 (11.3)	26 (8.8)	38 (13.9)	0.056

Hs-TnT - high-sensitivity troponin T; SOFA - Sequential Organ Failure Assessment; SCr - serum creatinine; AKI - acute kidney injury; KDIGO - Kidney Disease: Improving Global Outcomes; ICU - intensive care unit. *Ten patients did not use heparin during hospitalization; 218 patients used only one heparin dose regimen (prophylactic, intermediate dose or full dose); 151 patients used only the prophylactic heparin dose regimen; 50 patients used only an intermediate heparin dose regimen; 17 patients used only a full heparin dose regimen; 240 patients used more than one heparin dose regimen: 154 patients used prophylactic and intermediate doses, 51 patients used prophylactic and full doses, 35 used intermediate and full doses; 99 patients used the three heparin dose regimens; †the highest value in the first 3 days after intensive care unit admission. Results expressed as n (%) or medians and interquartile ranges (25th and 75th percentiles).

Table 4S - Cardiovascular events* of patients with COVID-19 according to the development of myocardial injury

	Overall<br>n = 567	hs-TnT ≤ 14ng/L<br>n = 294	hs-TnT > 14ng/L<br>n = 273	p value
Clinical outcomes				
Cardiovascular event	113 (19.9)	36 (12.2)	77 (28.2)	< 0.001
No cardiovascular event	454 (80.1)	258 (87.8)	196 (71.8)	< 0.001
1 cardiovascular event	91 (16.0)	30 (10.2)	61 (22.3)	< 0.001
≥ 2 cardiovascular events	22 (3.9)	6 (2.0)	16 (5.9)	0.019
Type of first cardiovascular event (venous/arterial/heart failure or arrhythmia)				
Venous event	50 (8.8)	22 (7.5)	28 (10.3)	0.245
Arterial event	23 (4.1)	5 (1.7)	18 (6.6)	0.003
Heart failure or arrhythmia	40 (7.1)	9 (3.1)	31 (11.4)	< 0.001
Time (days) from ICU admission to cardiovascular event	12 (7 - 20)	12 (8 - 19)	12 (5 - 20)	0.143
Early cardiovascular event (first week after ICU admission)	68 (12.0)	22 (7.5)	46 (16.8)	0.001
Late cardiovascular event (more than 7 days after ICU admission)	45 (7.9)	14 (4.8)	31 (11.4)	0.004
Type of cardiovascular event				
Deep vein thrombosis	24 (4.2)	11 (3.7)	13 (4.8)	0.547
Pulmonary embolism	36 (6.3)	13 (4.4)	23 (8.4)	0.051
Stroke	7 (1.2)	3 (1.0)	4 (1.5)	0.716
Myocardial infarction	11 (1.9)	0 (0.0)	11 (4.0)	0.001
Acute limb ischemia (inferior)	11 (1.9)	4 (1.4)	7 (2.6)	0.299
Acute limb ischemia (superior)	0 (0.0)	0 (0.0)	0 (0.0)	-
Mesenteric ischemia	3 (0.5)	1 (0.3)	2 (0.7)	0.611
Heart failure	10 (1.8)	4 (1.4)	6 (2.2)	0.533
Arrhythmia				
All arrhythmias	37 (6.5)	7 (2.4)	30 (11.0)	< 0.001
Supraventricular arrhythmia (atrial flutter; atrial fibrillation; supraventricular tachycardia)	30 (5.3)	7 (2.4)	23 (8.4)	0.001
Ventricular arrythmia (ventricular tachycardia; ventricular fibrillation)	8 (1.4)	0 (0.0)	8 (2.9)	0.003
Atrioventricular block	1 (0.2)	0 (0.0)	1 (0.4)	0.481

Hs-TnT - high-sensitivity troponin T; ICU - intensive care unit. *Cardiovascular event: the composite of deep vein thrombosis; pulmonary embolism; stroke; myocardial infarction; acute limb ischemia (inferior or superior); mesenteric ischemia; heart failure; arrhythmia (supraventricular [atrial flutter; atrial fibrillation; atrial tachycardia]; ventricular [ventricular tachycardia; ventricular fibrillation]; atrioventricular block). Results expressed as n (%) or medians and interquartile ranges (25th and 75th percentiles).
